# Molecular phylogeny of beetle associated diplogastrid nematodes suggests host switching rather than nematode-beetle coevolution

**DOI:** 10.1186/1471-2148-9-212

**Published:** 2009-08-24

**Authors:** Werner E Mayer, Matthias Herrmann, Ralf J Sommer

**Affiliations:** 1 Max Planck Institute for Developmental Biology, Department of Evolutionary Biology, Spemannstr. 37, 72076 Tübingen, Germany

## Abstract

**Background:**

Nematodes are putatively the most species-rich animal phylum. They have various life styles and occur in a variety of habitats, ranging from free-living nematodes in aquatic or terrestrial environments to parasites of animals and plants. The rhabditid nematode *Caenorhabditis elegans *is one of the most important model organisms in modern biology. *Pristionchus pacificus *of the family of the Diplogastridae has been developed as a satellite model for comparison to *C. elegans*. The Diplogastridae, a monophyletic clade within the rhabditid nematodes, are frequently associated with beetles. How this beetle-association evolved and whether beetle-nematode coevolution occurred is still elusive. As a prerequisite to answering this question a robust phylogeny of beetle-associated Diplogastridae is needed.

**Results:**

Sequences for the nuclear small subunit ribosomal RNA and for 12 ribosomal protein encoding nucleotide sequences were collected for 14 diplogastrid taxa yielding a dataset of 5996 bp of concatenated aligned sequences. A molecular phylogeny of beetle-associated diplogastrid nematodes was established by various algorithms. Robust subclades could be demonstrated embedded in a phylogenetic tree topology with short internal branches, indicating rapid ancestral divergences. Comparison of the diplogastrid phylogeny to a comprehensive beetle phylogeny revealed no major congruence and thus no evidence for a long-term coevolution.

**Conclusion:**

Reconstruction of the phylogenetic history of beetle-associated Diplogastridae yields four distinct subclades, whose deep phylogenetic divergence, as indicated by short internal branch lengths, shows evidence for evolution by successions of ancient rapid radiation events. The stem species of the Diplogastridae existed at the same time period when the major radiations of the beetles occurred. Comparison of nematode and beetle phylogenies provides, however, no evidence for long-term coevolution of diplogastrid nematodes and their beetle hosts. Instead, frequent host switching is observed. The molecular phylogeny of the Diplogastridae provides a framework for further examinations of the evolution of these associations, for the study of interactions within the ecosystems, and for investigations of diplogastrid genome evolution.

## Background

Nematodes constitute one of the largest animal phyla, both in number of species and in number of individuals [1]. They occur in terrestrial, freshwater and marine environments as free-living species, as well as parasites of plants and animals in a high variety of ecosystems. A consistent evolutionary framework using molecular data was provided by Blaxter et al. [2] and expanded by Holterman et al. [3].

Nematodes represent an attractive system for evolutionary developmental biology, given that some species can easily be cultured in the laboratory. One such system is the diplogastrid nematode *Pristionchus pacificus*, which has been established as a laboratory model organism and satellite system to the rhabditid nematode *Caenorhabditis elegans *[4,5]. Molecular, genetic, and genomic toolkits have been developed and the *P. pacificus *genome has recently been sequenced [6]. A detailed genetic comparison of sex determination, vulva and gonad development reveals how developmental processes changed between *P. pacificus *and *C. elegans *[7-10]. Such studies can elucidate the mechanisms by which developmental novelty is generated [11].

*P. pacificus *and *C. elegans *do not only differ in their development but also in their ecology. The ecological niche occupied by *Pristionchus *shows a close association to beetles, preferentially to members of the Scarabaeidae, but also to the Colorado potato beetle, a chrysomelid [12-14]. Important factors for host recognition appear to be provided by chemical cues emanated from the beetles [15,16]. For example, *P. pacificus *was frequently obtained from the oriental beetle *Exomala orientalis *in Japan and the United States and is the only *Pristionchus *species that is attracted to the oriental beetle sex pheromone *(Z)*-7-tetradecen-2-one in olfactory studies [14].

Evolutionary and comparative studies, like the ones mentioned above, require a robust phylogeny. A phylogenetic tree of the genus *Pristionchus *served as a first step in providing such a framework [17]. However, a full phylogenetic consideration has to involve higher taxa and has to place the genus *Pristionchus *into i) the family of the Diplogastridae and ii) more generally, clade V nematodes. While Kiontke et al. [18] convincingly placed the Diplogastridae as a monophyletic clade within the family of the Rhabditidae, few attempts have been made to provide a molecular phylogeny of the Diplogastridae family. Ultimately, such studies are necessary to elucidate macroevolutionary traits and patterns (such as characteristics of vulva developmental, olfaction-guided behaviour, acquisition of new genes or gene functions and others) for both, the comparison of taxa within the Diplogastridae (*i.e*. the origin of *Pristionchus *and *Pristionchus *traits) and the comparison of *Pristionchus *to *C. elegans*.

Based on morphological characters, the Diplogastridae have been revised and reclassified into 28 genera by Sudhaus and Fürst von Lieven [19]. Many of the diplogastrid genera are fully associated with beetles, *i.e*. they are carried by beetles or live in habitats closely linked to beetle activity. In some other genera, a single species shows a nematode-beetle association. Molecular phylogenies involving the deeper level phylogeny of the Diplogastridae are limited. To our knowledge, only two attempts have been made by Holterman et al. and Kiontke et al., who placed four diplogastrid taxa as a monophyletic clade within the family Rhabditidae [3,18].

In order to understand the ecosystems, in which *Pristionchus *evolved and to provide a framework for behavioural and comparative genomics studies we set out to reconstruct robust phylogenetic relationships of beetle-associated diplogastrids. We collected molecular data from 12 ribosomal protein genes representing 14 diplogastrid genera. In total, we used 2148 parsimony-informative characters for phylogenetic analyses. Our data identify four distinct subclades within the Diplogastridae. Furthermore, our dataset suggests that the observed patterns of beetle associations is driven by host switching rather than nematode - beetle coevolution.

## Results

### Collection of nematodes

Seventeen nematode taxa, representing 14 diplogastrid genera, plus one outgroup, *Rhabditoides inermis*, were selected according to whether some, or all of their members, showed direct association with beetles or shared the same habitat (Table 1) [19-24]. Whenever possible, established and characterized nematode lines were chosen. For the remaining taxa, host beetles were collected in their respective habitat and nematodes carried by them were isolated as described by Herrmann et al. [12]. Emerging nematodes were assigned to genus level by Nomarski microscopy following the keys given by Andràssy and Sudhaus and Fürst von Lieven [19,21]. Where possible, established strains were kept as growing cultures in the laboratory and as permanent frozen stocks.

**Table 1:** Origin of nematode specimens

Nematode taxon	**Strain**^1^	Host taxon/soil	Location	**Lab**^2^	Reference
*Acrostichus *sp.	RS5083	soil	Breitenholz, Germany	RS	this study
*Diplogasteroides magnus*	RS5440	*Nosodendron fasciculare *(Nosodendridae)	Stuttgart, Germany	RS	this study
*Diplogasteroides magnus*	RS1983	*Melolontha melolontha *(Scarabaeidae)	Tübingen, Germany	RS	this study
*Diplogasteroides *sp.	RS5444	*Holotrichia *sp. (Scarabaeidae)	Kobe, Japan	RS	this study
*Diplogastrellus gracilis*	SB306		Sweden	SB	
*Koerneria *sp.	RS1982	*Melolontha melolontha *(Scarabaeidae)	Usedom, Germany	RS	[17]
*Koerneria sudhausi*	SB413		Israel	SB	[25]
*Micoletzkya *sp.	-	*Ips typographus *(Curculionidae)	Gotha, Germany	RS	this study
*Mononchoides *sp.	RS5441	*Geotrupes *sp. (Scarabaeidae)	Corsica, France	RS	this study
*Myctolaimus *sp.	RS5442	*Prioninae indet*. (Cerambycidae)	Ghana	RS	this study
*Neodiplogaster *sp.	-	*Pissodes pini *(Curculionidae)	Göttingen, Germany	RS	this study
*Oigolaimella attenuata*	SB353	*Reticulitermes lucifugus*	Corsica, France	SB	[26]
*Pristionchus pacificus*	PS312	*soil*	Pasadena, USA	PS	[4]
*Pristionchus uniformis*	RS0141	*Melolontha melolontha *(Scarabaeidae)	Menz, Germany	RS	[12]
*Pseudodiplogasteroides *sp.	SB257	rotten cacti	Tucson, Arizona, USA	SB	[24]
*Rhabditidoides *sp.	RS5443	*Geotrupes *sp. (Scarabaeidae)	Tübingen, Germany	RS	this study
*Rhabditoides inermis*	SB328			SB	
*Tylopharynx *sp.	-	*Geotrupes *sp. (Scarabaeidae)	Corsica, France	RS	this study

^1^Nematodes without strain designation could not be kept in culture.

^2^Lab abbreviations: RS, Ralf J. Sommer, Tübingen; SB, Walter Sudhaus, Berlin; PS, Paul Sternberg, Pasadena.

### Identification and estimation of relationship of diplogastrid genera by *SSU *analysis

**Figure 1:**
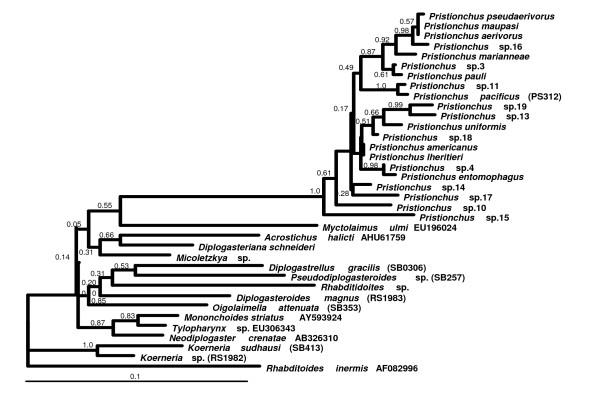
**Maximum likelihood tree of diplogastrid *SSU *sequences**. Sequences of 21 *Pristionchus *species ([17] and unpublished data) and 14 diplogastrid *SSU *sequences retrieved from the GenBank database or obtained from isolates described here were aligned manually with *Rhabditoides inermis *as outgroup. A maximum likelihood tree was reconstructed using the phylogeny.fr server [38]. GenBank accession codes are shown at taxon labels where available, strain designations are shown in parentheses. Bootstrap support values are indicated at nodes.

To obtain a first set of molecular phylogenetic characters a 1 kb-fragment of the nuclear *SSU *was amplified and 0.5 kb of the sequence at the 5' end was determined as described in Mayer et al. [17]. The sequences of all diplogastrid samples were aligned together with diplogastrid nuclear *SSU *sequences from the GenBank database [25]. *Rhabditoides inermis *served as outgroup. A maximum likelihood tree was constructed using the phylogeny.fr server [26]. All species within a genus, *i. e*. all *Pristionchus *species and both *Koerneria *species, were placed in distinct clusters separated from other genera by long branches (Figure 1). However, internal nodes connecting different genera received only moderate to low support values. Thus an extended dataset is required to better resolve the phylogeny. To avoid inferring the genus tree from a single, although in itself possibly robust, gene tree we opted to acquire a dataset of ribosomal protein gene-derived ESTs representing major parts of the genome.

### Phylogenetic analyses

Single ML gene trees for the 12 ribosomal protein genes and the *SSU *segment were obtained and a consensus tree was generated (Additional file 1). Internal node topologies in the derived consensus tree were recovered at a low percentage only due to incongruent single gene trees. However, major clades could be distinguished as has already been indicated in the initial *SSU*-based tree. Three major clades were identified. The genera *Diplogasteroides*, *Pseudodiplogasteroides*, *Diplogastrellus*, and *Rhabditidoides *clustered in one group; the second group consisted of *Mononchoides*, *Neodiplogaster*, and *Tylopharynx *and the third group encompassed *Diplogasteriana*, *Acrostichus*, and *Micoletzkya*. Individual species of a genus always grouped together. A conspicuous finding in the phylogenetic tree is that both members of the genus *Koerneria *adopted a basal position to the other diplogastrids (Additional file 1).

For a combined analysis of all sequenced genes, the single gene alignments of the ribosomal protein and the *SSU *genes were concatenated to yield a final dataset of 18 aligned sequences of 5996 bp in length. For the analyses, 216 sites at gapped positions were excluded. Of the remaining 5750 sites, 2899 were constant, 703 were variable but parsimony-uninformative, and 2148 were parsimony informative.

**Figure 2:**
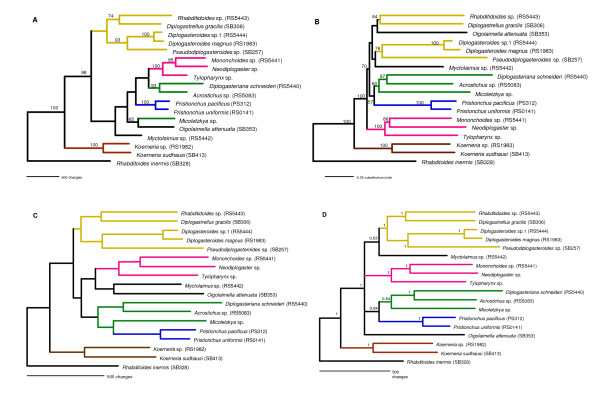
**Phylogenic relationships of Diplogastridae based on concatenated *SSU *and ribosomal protein genes**. The phylogenetic trees of 17 diplogastrid taxa were reconstructed from 5,996 bp of concatenated ribosomal protein CDS and *SSU *sequences. Coloured branches denote supported diplogastrid clades. Robustness of the tree topologies was evaluated by 1000 bootstrap replications. The support values are shown at the nodes. The trees were rooted by *Rhabditoides inermis *as outgroup. **A**. Maximum parsimony tree obtained by the heuristic search algorithm with the help of the PAUP*4.0b10 software [28]. Multistate characters were interpreted as polymorphisms, gaps as fifth state. **B**. Neighbour-joining tree. The tree was reconstructed using the BIONJ algorithm [42] and ML distances. **C**. Maximum likelihood tree. The codons were partitioned to three sites, corresponding to the GTR+SS model and the phylogenetic relationships were reconstructed by the heuristic search algorithm. **D**. Phylogenetic tree obtained by Bayesian inference using the Bayesian Estimation of Species Trees (BEST) software [35] applying gene partition. The analysis was run for 2,000,000 generations with a burnin of 500,000 generations. Tree sampling frequency was 1 in 100 generations. Numbers at nodes indicate posterior probabilities. The same tree topology was obtained using MrBayes 3.1.2 (Additional file 1) [29].

MP, NJ and ML trees were reconstructed as described (see Material and Methods)[27,28]. Figure 2 displays the resulting trees rooted by *Rhabditoides inermis *as outgroup. Again, conspicuious clustering of (1) the genera *Diplogasteroides*, *Pseudodiplogasteroides*, *Diplogastrellus*, and *Rhabditidoides*, of (2) the genera *Mononchoides*, *Neodiplogaster*, and *Tylopharynx*, and of (3) *Diplogasteriana *and *Acrostichus *was observed. In the NJ tree (Figure 2B), the first clade, though, also includes *Oigolaimella*. The topological arrangement of these clusters, however, received low statistical support. The position of four genera remained undetermined. These were *Pristionchus*, *Micoletzkya*, *Oigolaimella*, and *Myctolaimus*.

### Bayesian inference

Additional support for the deduced phylogenetic relationship was obtained by Bayesian inference. The complete concatenated dataset was evaluated by the MrBayes 3.1.2 software, applying the GTR+I+G substitution model (Additional file 1), and by its modification as in the BEST software, which estimates the joint posterior distribution of gene trees and species (Figure 2D) [29,30]. Both trees showed congruent topologies. Strong support by posterior probability values of 1.0 was given to the clades defined by the MP, NJ, and ML procedures. While the phylogenetic position of *Myctolaimus *and *Oigolaimella *still remained elusive in those analyses, Bayesian inference clustered the remaining four taxa *Pristionchus, Micoletzkya *and *Diplogasteriana*/*Acrostichus*. The posterior probability values, however, were only significant for the *Diplogasteriana*/*Acrostichus *pair. *Pristionchus *appears to be most divergent, next to *Micoletzkya*. *Acrostichus *and *Diplogasteriana *consistently group with high support values in all reconstructed trees. The early divergence of the genus *Koerneria *from other diplogastrids was highly supported. The lack of topological resolution and short branch lengths at internal nodes observed in all tree reconstruction algorithms points to an inherent property of this (and probably similar) datasets, which is caused by rapid ancestral radiations and incomplete assortment of ancestral polymorphisms.

## Discussion

### Phylogenetic species tree of the Diplogastridae

Here we present a first comprehensive molecular phylogeny of beetle-associated diplogastrid nematodes providing a framework for the investigation of nematode-beetle interactions and the interplay of ecological factors and nematode evolution. The Diplogastridae include more than 300 species of free-living or beetle-associated nematodes, which are grouped in 28 genera [19,20]. They are a monophyletic clade placed within the paraphyletic rhabditids [18]. The sister group to the diplogastrids is formed by the eurhabditids, which encompass *Caenorhabditis*, *Oscheius *and others [18].

For the purpose of assessing phylogenetic relationships, we choose representative species of the diplogastrid genera for molecular analyses. Phylogenetic species trees can be derived from single gene trees if speciation events are well separated in time. In general, however, this premise cannot be assumed and true species trees may differ from gene trees [31,32]. Thus, to expand the dataset, multiple genes are needed for a robust phylogenetic reconstruction. Phylogenetic species trees from multi-gene data can be inferred in two different ways. They can either be constructed from the consensus of separately generated gene trees or from an alignment of concatenated multiple gene sequences is used to infer the species tree. The latter was shown to perform better in yielding more accurate trees in a test situation [33]. Here, we applied both procedures, but chose to focus on the concatenation approach since the phylogenetic information content of the single gene sequences was limited.

### A molecular phylogeny of beetle-associated diplogastrid nematodes

Although the topologies of the phylogenetic trees produced by the different algorithms were not always congruent, four fundamental features can be extracted (Figure 2). Firstly, species of the same genus always clustered together, as shown by the genera *Pristionchus*, *Koerneria*, and *Diplogasteroides*, for which two geographically distinct species each were included in the analysis. The two divergent species of *Koerneria *displayed the highest intra-genus distance, whereas *Pristionchus *and *Diplogasteroides *species from Europe and North America or Japan, respectively, grouped together more closely. This feature is consistent with the morphological separation of the two subclades within *Koerneria*, as already discussed by Fürst von Lieven [23]. Secondly, terminal branches were exceedingly long while branches separating internal nodes with low support values were very short or collapsed completely. Although most beetle-associated diplogastrid genera were represented in the analyses, increased taxon sampling including non-beetle associated diplogastrids, might resolve some of those internal nodes. Overall, however, this indicates rapid initial diversification and speciation events separated by short divergence times in relation to the persistence of the extant taxa. Genealogies of individual genes may contribute to misleading species tree topologies if polymorphisms in ancestral populations of high effective population sizes are incompletely sorted during speciation events in close succession [32]. The long persistence of the extant genera resulted in accumulation of multiple substitutions in terminal branches. Terminal taxa, therefore, can be expected to be rich in homoplasic characters due to parallel sequence evolution or evolutionary convergence. The resulting long-branch attraction effects interfere with the clear resolution of internal nodes. Thirdly, several monophyletic clades with high support from bootstrapping or from posterior probabilities became evident from the comparison of various trees. The first clade encompassed *Diplogasteroides *with *Pseudodiplogasteroides *as its putative sister genus, *Diplogastrellus*, and *Rhabditidoides*. The second clade included *Mononchoides*, *Neodiplogaster *and *Tylopharynx*. The third group combined *Acrostichus *and *Diplogasteriana*. The inclusion of *Micoletzkya *and *Pristionchus *in this group occurs with posterior probabilities of ≥ 0.8 and thus remains unresolved. The taxa *Myctolaimus *and *Oigolaimella *could not be linked firmly to any of the above clades. Fourthly, the genus *Koerneria*, which was in close relationship to *Pristionchus *in previous studies, assumes a basal position to all diplogastrid nematodes in this molecular analysis [19,20]. This topology is also indicated in the molecular phylogeny of Kiontke et al. [18].

### Comparison of molecular and morphological phylogenies

There is a fairly good agreement between the phylogeny of Diplogastridae as proposed by Sudhaus and Fürst von Lieven based on apomorphic morphological characters and the molecular phylogeny presented here [19]. This example strongly supports the fact that morphological and molecular phylogenies should - under ideal conditions - result in similar findings. If morphological characters are well chosen and molecular characters are informative and substantial, homoplasy should be reduced, thus resulting in similar phylogenies.

The only major discrepancy in the case of the Diplogastridae is the genus diverging first from the stem species of Diplogastridae (see above). While in the morphological tree this position is taken by *Pseudodiplogasteroides*, in the molecular tree it is occupied by *Koerneria*. As a consequence, the molecular analysis places *Pseudodiplogasteroides *as the sister taxon to *Diplogasteroides*. The same well-supported clade also unexpectedly harbours the genus *Diplogastrellus*, which appears to be closer to *Acrostichus *in a yet unresolved relationship following Sudhaus and Fürst von Lieven [19]. The latter, on the other hand, is the sister taxon to *Diplogasteriana *in the molecular tree.

Although positioned distinctively on the molecular phylogenetic tree, *Pristionchus *and *Koerneria *share a number of morphological characters, such as a dimorphism in the buccal cavity, the shape and arrangement of denticles, overall body shape, and a prominent ripping of the cuticle [19]. These features could have evolved in two different ways. First, the shared characters may have been present in the stem species followed by subsequent loss in genera other than *Pristionchus *and *Koerneria*. This scenario is, however, unlikely since multiple independent character losses were required. The second, more likely possibility assumes parallel or convergent evolution. Given the long terminal branches separating the genera, independent convergent gain of advantageous characters is easily conceivable.

### Nematode-beetle associations: Coevolution or host switching?

Is there a correlation between the rapid ancestral diversification of the diplogastrid lineages and their subsequent evolution with the phylogeny of their coleopteran hosts? The beetles appeared around 285 million years ago (mya) followed by radiations of suborders [34-36]. The minimum age of the Scarabaeoidea and the Chrysomeloidea, which are major hosts for Diplogastridae, were both estimated to 150 mya, whereas their last common ancestor lived more than 236 mya [36]. Dieterich et al. estimated the divergence of the *Pristionchus pacificus *from the eurhabditid genus *Caenorhabditis *to a time range of 280-430 mya [6]. The ancestral radiations of Diplogastridae thus occurred in a time period that overlaps with the diversification of beetles into major lineages. It is therefore conceivable that the initial radiation of the Coleoptera had influence on the diversification of diplogastrids by providing new ecological niches, to which the nematodes were adapting. If these interactions persisted for a long time, coevolution of the nematodes with the beetles should have shaped the evolutionary pattern of the diplogastrids and its traces should be detectable in phylogenetic tree topologies. Inspecting the monophyletic diplogastrid clades, such as *Mononchoides*, *Tylopharynx*, and *Neodiplogaster *for these features will provide clues to resolve this issue. *Mononchoides *is associated with scarab beetles, curculionids, and *Silphidae*, *Tylopharynx *is associated with scarabs, and *Neodiplogaster *with curculionid, buprestid and cerambycid beetles. Likewise, in a second monophyletic group, *Diplogasteroides *and *Diplogastrellus *occur on curculionids and cerambycids, *Pseudodiplogasteroides *on cerambycids, and *Rhabditidoides *on Scarabaeidae. Following the comprehensive beetle phylogeny provided by Hunt et al. the Cucujiformia, which encompass cerambycid, and chrysomelid beetles, originated 236 mya, well separated from Scarabaeoidea [36]. Thus, members of the same monophyletic diplogastrid clade or even the same genus are associated with beetles of ancient divergence, refuting the possibility of a long-term coevolutionary relationship. Together, these data support host switching rather than coevolution as mechanism to explain the observed patterns of diplogastrid - beetle associations.

A case of recent host switching of a European species to an American host is given by *Pristionchus uniformis *[13,17]. *Pristionchus *dauer larvae are generally found on scarab beetles but *Pristionchus uniformis *also infests at high frequencies the Colorado potato beetle, a chrysomelid beetle, both in Europe and in North America [12,13]. The nematode populations on the two hosts species are genetically undistinguishable indicating recent and repeating events of host changes. The evolution and population genetics are currently under investigation (Isabella d'Anna, W.E.M. and R.J.S., unpublished observations).

### Phylogeny: A further mark for genome evolution

Sequencing the whole genome of an organism results in data that can only be interpreted in the context of a robust phylogenetic framework, including other related taxa. The genome of *P. pacificus *has recently been sequenced and the analysis of its genetic composition identified homologous nematode-specific genes, but also a remarkable number of features not shared with *C. elegans *or other available nematode genomes [6]. Firstly, the *P. pacificus *genome contains 23,500 predicted protein-coding genes, a substantial amount of which does not show sequence similarities to other metazoan genes. Secondly, the *P. pacificus *genome contains a massive expansion of genes predicted to be involved in the detoxification of xenobiotics, such as cytochrome P450 enzymes and ABC transporters. Thirdly, a conspicuous set of genes encoding cellulases or glycosylhydrolases is apparent, which has no counterpart in other nematodes but is most similar to corresponding genes in *Dictyostelium *or other microbes. The acquisition of these and several other genes could only be explained by horizontal gene transfer. With phylogenetic information on the taxa involved, as presented here for subclades of the Diplogastridae the analyses of horizontal gene transfer events in diplogastrid genomie evolution can be studied in greater detail in the future.

## Conclusion

Molecular data from 14 diplogastrid beetle-associated genera were collected and used to obtain some robust phylogenetic relationships. The following conclusions can be drawn. Firstly, whereas terminal branches of the resulting phylogenetic tree are long, indicating long-term accumulation of substitutions, internal branches, separating four robust clades are short. These are (1) *Diplogasteroides*, *Pseudodiplogasteroides*, *Diplogastrellus*, and *Rhabditidoides*, (2) *Mononchoides*, *Neodiplogaster*, and *Tylopharynx*, (3) *Acrostichus *and *Diplogasteriana*, and (4) both *Koerneria *subclades. Internal nodes could not be resolved with high support, indicating ancient rapid radiation and incomplete gene lineage sorting. Secondly, although the Diplogastridae-beetle association appears to be ancient, no long-term coevolution is evident. Instead, there are indications of frequent host-switching of diplogastrid nematodes. Thirdly, the present phylogeny of the Diplogastridae provides a framework for further investigations of diplogastrid-beetle associations and the ecological influences shaping their evolution. Fourthly, this study is of important interest for a deeper search into the origins of observed horizontal-gene transfer events in nematodes and for the phylogenetic interpretation of diplogastrid genome evolution.

## Methods

### Sources of diplogastrid nematodes

The origin of nematode strains or beetles with associated nematodes is listed in Table 1.

### Genus identification of diplogastrid specimens

#### Morphology

Sacrificed beetles were placed onto 6 cm NGM agar plates [12]. Over a period of one to two weeks emerging nematodes were determined to family level by using a dissecting scope (Zeiss Stemi 2000) and classified to genus level with a microscope (Zeiss Axioplan2) using keys from Andràssy and Sudhaus and Fürst von Lieven [19,21].

### Molecular identification of genera

The nuclear small subunit rRNA gene sequence (*SSU*) was employed for molecular characterization of nematode samples [17,37]. To this end, genomic DNA from single nematodes was released using the NaOH lysis procedure described by Floyd et al. [37] and as in Herrmann et al. [12]. One microliter of this extract was used for subsequent polymerase chain reaction (PCR) with primers SSU18A (5'-AAAGATTAAGCCATGCATG-3') and SSU26R (5'-CATTCTTGGCAAATGCTTTCG-3') [2,37] as in Herrmann et al. to amplify a 1 kb fragment [12]. A 500 bp segment of the 5'-terminal end of the *SSU *was directly sequenced using the primer SSU9R (5'-AGCTGGAATTACCGCGGCTG-3') and the Big Dye terminator sequencing mix (Applied Biosystems, Darmstadt, Germany).

### Ribosomal protein genes in expressed sequence tag (EST) libraries

#### EST library and data acquisition

EST libraries were prepared from cultures of diplogastrid nematodes as described by Mayer et al. [17]. The selected strains were *Diplogasteroides magnus *RS1983, *Diplogastrellus gracilis *SB306, *Acrostichus *sp. RS5083, and *Rhabditoides inermis *SB328. *Rhabditoides inermis *was chosen as closest non-diplogastrid outgroup according to Kiontke et al. and in agreement with *SSU*-based phylogenetic trees (Figure 1) [18]. The dataset was combined with EST data previously obtained from *Pristionchus *species and *Koerneria *sp. RS1982 [17]. Twelve abundantly expressed monoallelic ribosomal protein genes were selected from this dataset (Table 2). RT-PCR with gene-specific primers (Additional file 2: Table S1) served to complete the ribosomal protein gene dataset for these six taxa and to obtain the corresponding sequences from a second *Diplogasteroides *species from Japan, a divergent *Koerneria *species (*K. sudhausi*), and from eight additional diplogastrid genera encompassing those collected from beetles (Table 1) [4,12,17,24-26]. Plasmid DNA from about 400 single EST clones each was extracted using the QIAprep 96 Turbo BioRobot Kit (Qiagen) and the inserts were sequenced from the 5' end using the SL1-specific primer BJ234 (5'-GGTTTAATTACCCAAGTTTGAG-3').

**Table 2:** Ribosomal protein genes used in the study

Gene	Omitted nucleotides at 5' end	**Size of included coding sequence (in bp)**^1^	Encoded amino acids	Chromosomal position of homologue in *C. elegans*
*rpl-2*	21	762	253	V:25.00
*rpl-6*	0	768	243	III:-0.77
*rpl-9*	18	552	183	III:-0.66
*rpl-10*	33	612	203	II:0.71
*rpl-14*	12	402	133	I:3.85
*rpl-23*	15	408	135	III:-1.45
*rpl-29*	0	189	62	IV:10.90
*rpl-35*	24	351	115	III:-0.54
*rps-7*	0	591	196	I:4.94
*rps-14*	24	435	144	III:-0.78
*rps-27*	0	258	85	V:-20-01
*rps-28*	12	189	62	IV:-16.37

^1 ^including stop codons and alignment gaps

^2 ^stop codon in *Tylopharynx *sp. codon 14 was disregarded

#### EST analysis and gene-specific primer design

The proteins encoded by the SL1-transspliced genes in the initial EST screen were identified by BLASTX searches of WormBase and the non-redundant database at GenBank [22,38]. Sequences of selected ribosomal protein genes were aligned manually with the help of the Seqpup 0.6f software for Macintosh [39]. Conserved sequence stretches from this data set and from subsequently generated additional data were chosen to design gene-specific generic RT-PCR primers (Additional file 2). The primers were checked for secondary structures and compatibility to the SL1-primer BJ234 and the oligo(dT)-containing primer RH5620 (5'-GAAGATCTAGAGCGGCCGCCCTTTTTTTTTTTTTTT-3') with the help of the OLIGO 4.0 software (MedProbe, Oslo, Norway).

#### Reverse transcription polymerase chain reaction (RT-PCR) of ribosomal protein genes

RNA was reverse transcribed into cDNA with the help of the Sensiscript reverse transcriptase kit (Qiagen, Hilden, Germany) and the primer RH5620. Complete transcripts were synthesized by RT-PCR in two overlapping fragments per gene as described in Mayer et al. [17]. Briefly, the SL1-specific primer BJ234 was used in combination with gene-specific antisense primers to obtain the 5' parts of the transcripts and the combination of RH5620 and the sense primers to obtain the 3' parts. The PCR was performed using the HotStar *Taq *Plus DNA polymerase kit (Qiagen) including 1× Q solution in the reaction mix. PCR conditions were initial activation of the enzyme at 95°C for 5 min, followed by 40 cycles of denaturation at 94°C for 30 sec, primer annealing at 50°C for 30 sec, and primer extension at 72°C for 3 min. The reaction was completed by incubation at 72°C for 10 min. PCR fragments were electrophoresed through an 1.5% agarose gel, purified using the Wizard SV gel purification kit (Promega) and sequenced directly using the respective gene-specific PCR primers. The new sequences described here have been deposited to the GenBank database and can be retrieved under the Accession Numbers [GenBank: GQ422155-GQ422358].

### Phylogenetic analyses

#### Nuclear small subunit ribosomal rDNA

Sequences obtained from diplogastrid specimens were aligned to diplogastrid *SSU *sequences retrieved from the GenBank database by the ClustalX software [40] and manually improved with the help of the Seqpup 0.6f software for Macintosh [39]. According to Kiontke et al. (2007) *Rhabditoides inermis *was included as closest related outgroup [18]. The best-fit substitution model and the parameter settings were determined by the Modeltest 3.7 software using the Akaike information criterion [27,41]. The parameters for the selected model (GTR+I+G) were as follows: Estimated base frequencies: A = 0.2751, C = 0.2094, G = 0.2433, T = 0.2722; substitution rates A-C = 0.9722, A-G = 1.9547, A-T = 2.1277, C-G = 0.5838, C-T = 3.7945, G-T = 1.0000; proportion of invariable sites I = 0.2117; gamma distribution shape parameter α = 0.4911.

Phylogenetic relationships between the diplogastrid taxa were assessed based on 471 bp of the *SSU *sequences with the help of the PAUP*4.0b10 software using maximum parsimony (MP, heuristic search, gaps as 5^th ^state), neighbour-joining (NJ, BioNJ algorithm with maximum likelihood distances) methods, and heuristic search using maximum likelihood (ML) as optimality criterion [28,42]. Bootstrap support values were obtained by 1000 replications using the neighbour-joining method. Additionally, the Phylogeny.fr server at http://www.phylogeny.fr/ was employed [23]. From the alignment, ambiguous regions (*i.e*. containing gaps and/or poorly aligned) were removed with Gblocks (v0.91b) using the following parameters:

-minimum length of a block after gap cleaning: 10

-positions with a gap in less than 50% of the sequences were selected in the final alignment if they were within an appropriate block

-all segments with contiguous nonconserved positions bigger than 8 were rejected

-minimum number of sequences for a flank position: 85%. The phylogenetic tree was reconstructed using the maximum likelihood method implemented in the PhyML program (v3.0 aLRT) [43]. The GTR substitution model was selected assuming an estimated proportion of invariant sites (of 0.232) and 4 gamma-distributed rate categories to account for rate heterogeneity across sites. The gamma shape parameter was estimated directly from the data (gamma = 0.440). Reliability for internal branch was assessed using the aLRT test (SH-Like).

#### Ribosomal protein genes

Ribosomal protein coding cDNA sequences from 17 diplogastrid nematodes and *Rhabditoides inermis *were aligned by the ClustalX software or aligned manually with the help of the Seqpup 0.6f software for Macintosh [39,40]. Coding segments were deduced by comparison to the orthologues in *C. elegans, C. briggsae *and *P. pacificus*. Genes were selected according to presence of clear orthologues in the *C. elegans *genome, being distributed to the different chromosomes, their ease of amplification by RT-PCR with the SL1 primer and the presence of conserved sites for generic primer design. Genes that were either duplicated in *Caenorhabditis *or appeared to be present in more than one copy in the diplogastrid sample were omitted from the analysis. Reliably obtained coding sequences (depending on the gene, from codons 1 to 12 through the termination codon) were concatenated and subjected to analysis by Modeltest 3.7 to determine the best-fit nucleotide substitution model for maximum likelihood analysis (ML) using the Akaike information criterion [27,41]. For parameter determination only diplogastrid sequences were used. The model settings were equivalent to the GTR+I+G model with 6 substitution types (A-C = 0.9147, A-G = 1.3858, A-T = 1.2732, C-G = 1.0849, C-T = 4.5242, G-T = 1.0000), assumed nucleotide frequencies of A = 0.245038, C = 0.329976, G = 0.264464, T = 0.160522, assumed proportion of invariable sites I = 0.363795, rates at variable sites following the gamma distribution with shape parameter α = 1.026951.

A consensus tree of 13 single gene trees was constructed using PAUP*4.0b10 [28]. The dataset encompassing 17 diplogastrids and *Rhabditoides inermis *as outgroup was used to infer single gene trees by heuristic searches for each of the 12 ribosomal protein genes and for the *SSU *sequences. Heuristic searches with NJ starting trees were conducted under the maximum likelihood optimality criterion.

Phylogenetic trees of concatenated sequences were constructed by four algorithms: Neighbour joining (NJ), maximum parsimony (MP), maximum likelihood analysis (ML), and Bayesian inference (BI). NJ trees using the BIONJ method [42,44], MP trees and ML trees were reconstructed by PAUP*4.0b10 [28]. Alignment gaps were eliminated from the analysis. The branch-swapping algorithm was set to tree-bisection-reconnection (TBR). Support for the tree topology was obtained by 1000 bootstrap replications using the NJ or MP algorithms [45]. Phylogenetic trees by Bayesian inference were obtained using two softwares, MrBayes 3.1.2 software for Macintosh [29] and BEST [30]. For the MrBayes 3.1.2 software the evolutionary model was set to GTR+I+G. The analysis was run for 2,100,000 generations with a burnin of 500,000 generations and a sampling frequency of 1 in 100 generations. In order to estimate the best species tree that fulfils the constraint that all divergences in gene trees have to occur prior to species divergences, the modification of MrBayes as given by the Bayesian Estimation of Species Trees (BEST) software was employed with set gene partitions and run for 2,000,000 generations with a burnin of 500,000 generations and a sampling frequency of 1 in 100 generations [30].

Exported trees in Newick format were visualized with the help of the FigTree v1.2 software [46].

## Authors' contributions

WEM designed and carried out all of the molecular experiments, performed all of the phylogenetic analysis and wrote the manuscript. MH carried out all of the fieldwork, isolated and identified the nematodes. RJS designed and coordinated the experiments and wrote the manuscript. The study was funded by the Max Planck Society. All authors read and approved the final manuscript.

## Supplementary Material

Additional file 1 **Additional phylogenetic trees of *SSU *and 12 ribosomal protein genes coding sequences**. A. Consensus tree of individual maximum likelihood trees of *SSU *and 12 ribosomal protein genes coding sequences. Trees of 13 separate genes were reconstructed by heuristic search using the PAUP*4.0b10 software and maximum likelihood settings, corresponding to the GTR+I+G substitution model as determined by the Modeltest 3.7 software. The consensus tree was obtained using the PAUP*4.0b10 software. The frequencies of node topologies found in individual gene trees are indicated. B. Tree obtained by Bayesian inference using the MrBayes 3.1.2. software [34]. The evolutionary model was set to GTR+I+G. The analysis was run for 2,100,000 generations, burnin was for 500,000 generations, sampling frequency was 1 in 100 generations. *Rhabditoides inermis *was included as outgroup. Click here for file

Additional file 2 **Oligonucleotides used in this study**. Oligonucleotides used for RT-PCR and sequencing are shown. Click here for file
