# Supplementary material for: Molecular phylogeny of beetle associated diplogastrid nematodes suggests host switching rather than nematode-beetle coevolution
Source: BMC Evol Biol. 2009 Aug 24;9:212. doi: 10.1186/1471-2148-9-212 (PMC2737313; doi:10.1186/1471-2148-9-212)
Supplement: Additional file 2 — Oligonucleotides used in this study. Oligonucleotides used for RT-PCR and sequencing are shown. [file 1471-2148-9-212-S2.doc]

Table S1. Ribosomal protein-specific oligonucleotides employed in the study

Primer name Gene Orientation Sequence (5’ –> 3’)

WM16739 rpl-2 sense ATGCACACYGGMCAGTTCRTSTACT

WM16740 rpl-2 sense TYCCYTCYGGAGCCAAGAAGGTC

WM18610 rpl-2 antisense ATRTGYTGSTGGTTWCCACCACC

WM18611 rpl-2 antisense TGRGGRTGYTCVACGGGGTTCA

WM15353 rpl-6 sense GCMGGYCGTCATAAGGGAAAGC

WM15354 rpl-6 antisense GGTTAGCCTGSTCTCCCTTCTT

WM15355 rpl-6 sense CYGGCMGACAYAAGGGAAAGC

WM15356 rpl-6 antisense GGTGTCCGGATGCGAAGATGTC

WM16154 rpl-6 antisense AGATGTTRGCCTGCTCTCCCTT

WM16155 rpl-6 antisense AGAKSTCRGCSTTCTCTCCCTT

WM16156 rpl-6 antisense AGATRTYRGCSTGRTCWCCCTT

WM16890 rpl-6 antisense GAGGCCTTGGTGTTCTTCTTCTG

WM18677 rpl-6 antisense CTGCTCGCCCTTCTTCGCCTTCT

WM21169 rpl-6 antisense GCGGGGACCTTGACGGACGAGATG

WM15357 rpl-9 sense GACTTCCGYCACCTCYACATGG

WM15358 rpl-9 antisense ANACRARCTGRATGTCRTTTCC

WM15359 rpl-9 antisense TGRAKRGAGAYGTTGATGGGGA

WM16151 rpl-9 sense GAYTTCCGYCAYCTSCAYATGG

WM16152 rpl-9 sense TYCGYGTBCGCAAGTGGTTCGG

WM8117 rpl-10 sense CTCGTCAAGAACTGCGGAAAGGA

WM8118 rpl-10 antisense AGRCGRACGGAGAAGAGGAARTC

WM8568 rpl-10 antisense GTAGGCACCACGCATTCCAGTC

WM9621 rpl-10 sense CCAACAAGTTCCTCGTCAAG

WM9622 rpl-10 antisense TCGGCTCTCATTCTCTCGTA

WM14810 rpl-10 sense AGTCKCGSTTCTGYMGTGGAGT

WM14811 rpl-10 sense TBGWRAAGAACTGCGGWAAGGA

WM14812 rpl-10 antisense CGTCCGGGGAACTTGAACTTGG

WM20617 rpl-10 sense CCAACAAATACCTCGTGAAAAAC

WM11060 rpl-14 antisense AATCTGTTTCTGGCYTGCTT

WM12126 rpl-14 sense TGGTAGAAATCGGYCGTGTSGT

WM12127 rpl-14 antisense AGTCTRATRGGGACCTTGAACT

WM8066 rpl-14 antisense CGRTCGAAGTCSGWGAGRGTVGA

WM8067 rpl-14 sense CAAGTTCAAGGTCCCMATYAGA

WM8898 rpl-14 antisense GCAGCDAGCTTCTTDGCCCA

WM16741 rpl-14 sense GGAARGCTYGCCACSATCRTCAAC

WM16742 rpl-14 antisense TGAACTTSGTSARCTGVAKGTCCT

WM16891 rpl-14 antisense TCTTGGTCTTCTGCTGGAGGTGA

WM16892 rpl-14 sense GCCAAGGGCGAGAACGAGGGYAA

WM20618 rpl-14 antisense GGTTTCTTTGCTGCTTGGCTCTCAT

WM10288 rpl-23 antisense GTCCCTTCATTTCACCCTTGTT

WM10289 rpl-23 sense TWCGTGGAAGACTYAACAGACT

WM12129 rpl-23 sense GCCGGAGCTCAGGAAGAAGGTC

WM12169 rpl-23 sense GCGTCCGTCAAGAAGGGCAAGC

WM14813 rpl-23 sense TYATSAACTGYGCCGACAACAC

WM14814 rpl-23 antisense CCCTTCATYTCKCCCTTGTTGT

WM7991 rpl-29 sense CASYCAYAACCAGAACCGCAAGGA

WM7992 rpl-29 antisense TCTTGTYGTGCTTCTTGGCGAA

WM15348 rpl-29 sense CGCCCACAAYCAGAAYMGGAAGG

WM15349 rpl-29 sense MASYCACAAYCAGAAYMGCAAGG

WM15350 rpl-29 antisense CATGCTRCCCTTCYTSGMGAA

WM16153 rpl-29 antisense AACCVAGRTTCTTSAKGAACTT

WM21170 rpl-29 sense GCCAAGTCCAAGAACGCCTCCAAC

WM10282 rpl-35 sense GATYAGAGTSGTCCGCAAGAAC

WM10283 rpl-35 antisense AGGTACTKCTTKCCCTTGTAGA

WM14817 rpl-35 sense AGAMSYTBGAGGARCARAAGAC

WM14818 rpl-35 antisense GSTCMKSYTTCTGKGTCTGGTT

WM16500 rpl-35 sense AGACYCTYGAAGAGCARAAGAA

WM16501 rpl-35 antisense CAGATCMGTMGGCTTGTAYTTC

WM18678 rpl-35 antisense CTCTGTGCGGCTCTCTGTTGCTT

WM18679 rpl-35 antisense CTTGACRACRATGCGGAGGTTGG

WM20619 rpl-35 sense GCCAAGATCAGAACCGTCCGCAAGA

WM21171 rpl-35 antisense GCATGGCGCGGGTCTTCTTGTAG

WM21173 rpl-35 antisense TGCGCCCTTCTTATGCCTTGAC

WM14819 rps-7 sense CCARCTYAAGCAGTTCCAGAAG

WM14820 rps-7 antisense AWCKSGGCCTCTTCTGCTTCTC

WM16502 rps-7 sense CCAACTGAAGCAGTTCCAGAA

WM16503 rps-7 antisense GAGCGRGGSGKCTKCTGCTTCT

WM20620 rps-7 sense ATCGTTGGCGTGAAGGAGGTTGA

WM20621 rps-7 antisense CTTCAACCTCCTTCACGCCAACGAT

WM20975 rps-7 antisense TGCCAGTGAGGTTCTTGTARATG

WM21174 rps-7 antisense ACGTTCATCTGCTGCGACTTGT

WM8113 rps-14 antisense GGRGTCTTNGTTCTRGTTCCTC

WM8114 rps-14 sense GCYCAYATYTTCGCYTCTTTCAA

WM8569 rps-14 antisense TTGATGTGCAGAGCGTTGATAC

WM14821 rps-14 antisense CACGRTCRGCCTTCACCTTCAT

WM16504 rps-14 sense GGGAAAGACWAAGGAGGAGCA

WM21172 rps-14 sense CCAGGCCAAGGAGGGTGAGAAC

WM7993 rps-27 sense AYCCYAAYTCSTTCTTCATGGA

WM7994 rps-27 antisense GGYTTARTGCTGCTTCTTYCTGAA

WM7995 rps-27 antisense AGTTTAGTGGGCCTTCTTTCTGAA

WM14822 rps-27 sense ACCCSAAYTCNTWCTTCATGGA

WM20977 rps-27 antisense CATTTAATGCTGCTTCTTGCG

WM12703 rps-27 antisense CATCCGACGCAGACGACGACAG

WM11061 rps-28 sense CGCACTGGATCYCAGGGACA

WM8815 rps-28 sense TCGCTAAGGYCACCAAGATCCT

WM8816 rps-28 antisense GGCCCTTRACGTTGCGGATGAT

WM15351 rps-28 sense CGCACYGGMTCYCAGGGACA

WM15352 rps-28 antisense GGCCCTTCACGTTGCGGATGAT

WM20622 rps-28 sense CAAGCTCGCTAAAGTGACCAAGA
